# Anxiety about getting fat may increase the number of binge-eating disorder symptoms

**DOI:** 10.1038/s41598-025-21879-6

**Published:** 2025-10-30

**Authors:** Karolina Lewandowska, Waldemar Klinkosz, Wojciech Styk, Justyna Iskra

**Affiliations:** 1 Institute of Psychology, The Cardinal Stefan Wyszyński University in Warsaw, 01-938 Warsaw, Poland; 2 Academic Laboratory of Psychological Tests, Medical University in Lublin, 20-059 Lublin, Poland; 3 Institute of Psychology, The John Paul II Catholic University of Lublin, 20-950 Lublin, Poland

**Keywords:** Eating disorders, Binge-eating disorder, Depression, Anxiety, Anxiety about getting fat, Psychology, Human behaviour

## Abstract

According to preliminary sources, general anxiety and anxiety about getting fat (AGF) are associated with the occurrence of binge eating disorder (BED). Still, there is little research in this area. The aim of the present study was to investigate anxiety, AGF, and depression in BED. The research focused on BED from two separate perspectives: the number of BED symptoms and the recurrence rate of BED episodes. Women (*n* = 103) were surveyed using a self-developed questionnaire evaluating the presence of BED symptoms, the Hospital Anxiety and Depression Scale (HADS), and the Body Mass Anxiety Scale (BMAS-20). Participants who met the criteria for BED reported elevated levels of anxiety and AGF. Significant differences were observed in the level of anxiety and AGF between groups with a lower and higher number of BED symptoms. Also, groups with mild, moderate, and severe BED were found to differ significantly in the level of depression symptoms. AGF was associated with a greater number of BED symptoms, suggesting it may contribute to symptom escalation.

## Introduction

Binge eating disorder (BED) is characterized by frequently recurring episodes of overeating accompanied by a subjective sense of loss of control over eating. The experience of having lost control is the main feature of a binge eating episode. Unlike bulimia, binge eating episodes do not involve compensatory behaviors aimed at preventing weight gain (e.g., regular vomiting, taking laxatives, or regularly engaging in intense physical exercise). Consumption of large amounts of food is not connected with the feeling of hunger, and it generates high psychological costs, giving rise to guilt, shame, self-devaluation, and general suffering associated with overeating. The episodes are also associated with at least three of the following symptoms: (a) eating much more rapidly than usual, (b) eating until feeling uncomfortably full, (c) eating large amounts of food despite not feeling physically hungry, (d) eating alone because of feeling embarrassed, and (e) feeling disgusted with oneself, depressed and/or guilty during and/or after the episode^1^. BED often leads to weight gain and obesity over time, but it is important to note that people with BED can also have a normal weight.

In this study, anxiety is understood as an emotional state characterized by feelings of tension related to worried thoughts, fear, or stress^1^. Depressive disorder is described in ICD-11 as recurrent episodes of depressed mood, decreased interest, and loss of pleasure, accompanied by other cognitive, behavioral, or neurovegetative symptoms that negatively affect the individual’s ability to function normally^2^.

Mood and anxiety disorders are among the most commonly observed comorbidities in binge eating disorder. Co-occurring symptoms of depression and anxiety disorders in eating disorders are associated with greater symptom severity and poorer treatment outcomes, especially in young women^3^. Another source suggests that the interaction of obesity, anxiety, and depression is a potential predictor of BED, with the strongest positive relationship found between BED and depression^4^. Depression is regarded as a risk factor for BED and a potential cause of this disease^5^. Numerous studies confirm the co-occurrence of anxiety with binge eating^6–8^, and with bulimia^9^. Anxiety disorders may contribute to engaging in disordered eating behaviors^10^.

Researchers distinguish several types of anxiety disorders commonly co-occurring with eating disorders^10^. One such type of anxiety is anxiety about getting fat (AGF). AGF is considered a core feature of eating disorders and an indicator of their severity^11,12^. It is associated with psychopathological behaviors in both bulimia and anorexia nervosa^13,14^. Binge eating disorder is the least well-described eating disorder in this respect, having been recognized as a distinct clinical syndrome relatively recently. However, there is preliminary evidence to suggest that anxiety about getting fat may be a predictor of bulimia and BED^15,16^. This is consistent with our previous work, in which we were able to show that women who binge eat experience high levels of anxiety about getting fat^17^. There is also evidence that anxiety and depression are strongly associated with AGF and that an increased body mass index (BMI) reinforces this relationship^18^.

Although there is evidence for the importance of anxiety about getting fat in the broader context of eating disorders, there is little research to establish the association of AGF with general anxiety disorders in binge eating disorder. This present study aimed to look for relationships among symptoms of depression, anxiety, and AGF in BED. We adopted two perspectives on BED based on the DSM-5. This classification proposes specific criteria for assessing BED based on the number of symptoms (at least three) and the occurrence of episodes (at least one per week for three months)^1^. This study aimed to examine whether assessing BED severity based solely on the number of reported symptoms would differ from assessing BED based on the frequency of recurrent binge eating episodes. In most studies, BED severity was determined based on a time-based criterion, referring to the frequency of recurrent binge eating episodes^19^. We hypothesized that greater severity and greater diversity of BED symptoms would be associated with higher levels of anxiety and depression. We also expected that depression, anxiety, and AGF would predict the severity as well as the diversity of BED symptoms.

## Materials and methods

### Procedure

Data were collected through an online survey on social media from support groups for binge eaters and in person using paper test sheets from healthcare institutions serving patients with overweight, obesity, and eating disorders. Only women who were patients in bariatric wards and obesity treatment clinics participated in the in-person testing. The battery consisted of a BED symptoms scale, the Hospital Anxiety and Depression Scale (HADS), the Body Mass Anxiety Scale (BMAS), and a personal data sheet. The study involved only adults. All participants received information about the purpose of the study and the survey procedures. Informed consent was obtained from all participants. The study was approved by the Research Ethics Committee of the Institute of Psychology at the Cardinal Stefan Wyszyński University in Warsaw (registration number: 19/2022).

### Participants

The sample included 103 women aged 18 to 66 (M = 33.76; SD = 9.59). The participants were patients from obesity treatment clinics, bariatric wards, and psychological clinics; some belonged to the Association of Overeaters Anonymous [Stowarzyszenie Anonimowych Jedzenioholików] or online support groups for binge eaters. Inclusion in the sample required meeting the criteria for BED^1^. Symptoms of BED were assessed using a DSM-5-based scale developed by the present authors. According to DSM-5 criteria, individuals with at least three symptoms of BED were included in the study. Participants currently undergoing BED treatment (pharmacological or psychotherapeutic) were excluded. Additionally, individuals diagnosed with schizophrenia or bipolar disorder were excluded.

### Instruments

BED Symptoms Scale. The scale developed by the present authors is based on the diagnostic criteria of the American Psychiatric Association’s DSM-5 classification of mental disorders. It is a self-report instrument with eight items regarding BED symptoms and one control item regarding compensatory behaviors (item 9). The participants reported the symptoms they had experienced over the last month. The aim of constructing the scale was to verify the presence and number of BED symptoms in each study participant, as well as to assess the degree of severity of their symptoms. The diversity of BED symptoms was measured as the number of symptoms checked off by respondents in the scale. The severity of BED symptoms was assessed based on the frequency of binge-eating episodes: (1) mild—1 to 3 episodes per week; (2) moderate—4 to 7 episodes per week; and (3) severe—8 or more episodes per week.

Hospital Anxiety and Depression Scale (HADS)^20^. HADS is a 14-item self-report measure divided into two subscales: HADS-Anxiety (HADS-A) and HADS-Depression (HADS-D), which are used to assess the levels of anxiety and depression, respectively. Each item is rated on a 4-point Likert scale (from 0 to 3 points). The score range is 0–21, and the higher the score, the higher the severity of anxiety and depressive symptoms. The Polish translation of the test is characterized by high reliability, both for the anxiety subscale (Cronbach’s alpha of 0.8 or higher) and the depression subscale (Cronbach’s alpha of 0.9 or higher).

Body Mass Anxiety Scale (BMAS-20)^21^. BMAS-20 is a self-report questionnaire that assesses body mass-related anxiety levels. Each item is rated on a 7-point scale from 1 (Does not concern me at all) to 7 (Fully concerns me). The original Polish version of BMAS-20 and its English translation demonstrate good psychometric properties (Cronbach’s alpha of 0.916 and 0.91, respectively).

### Statistical analysis

The statistical analyses were conducted using JASP 0.17. Correlation analysis was performed using Spearman’s rank correlation coefficient. The values of the variables were compared across the three BED severity groups (mild, moderate, and severe) using the Kruskal-Wallis test followed by Tukey’s post hoc tests. Cluster analysis was used in an exploratory way to identify potential subgroups differing in the diversity of BED symptoms. Although a two-step approach (hierarchical followed by k-means) was applied to increase stability, we acknowledge that the modest sample size limits the reliability of the solution. Therefore, the clustering results are presented as preliminary and hypothesis-generating rather than confirmatory. Based on the cluster analysis, the participants were divided into groups with a greater and a lower diversity of BED symptoms. In the next step, the values of the test variables were compared using the non-parametric Mann-Whitney U test when the assumptions of the parametric test were not met. The results were considered significant at *p* <.05. As the last step, a linear regression model was performed to determine the direction of the relationships between the variables.

Given the exploratory nature of the study, we decided not to apply conservative multiple testing corrections (e.g., Bonferroni) across all analyses. The main reason was to avoid inflating the risk of Type II error, which could obscure potentially meaningful associations in a relatively small and homogeneous sample. Nevertheless, we highlight the results that reached the lowest p-values (*p* <.001), as they are less susceptible to Type I error and more robust against potential correction procedures.

## Results

### Descriptive statistics and correlation analysis

First, a descriptive analysis of the results was performed. Table 1 displays the means and standard deviations for the variables examined.

**Table 1:** Descriptive statistics and normality test.

Variables	M	SD	Sk.	Kurt.	S-W	*p*	Min	Max
Age	33.77	9.65	1.06	1.20	0.93	0.001**	18	66
BMI	27.80	8.27	1.25	1.40	0.89	0.001***	17.63	56.97
Severity of BED symptoms	1.44	0.64	1.17	0.27	0.67	0.001***	1	3
Number of BED symptoms (0–8)	6.04	1.86	−0.78	−0.23	0.88	0.001***	1	8
Anxiety about getting fat (AGF)	51.47	14.80	−0.76	−0.15	0.93	0.001***	11	70
Anxiety	16.85	3.54	0.53	−0.65	0.94	0.00***	11	25
Depression	17.73	3.39	−0.07	−0.66	0.97	0.04*	9	24

Analyses were performed for *N* = 105 observations; S-W – Shapiro-Wilk test; * *p* <.05, ** *p* <.01, *** *p* <.001.

As a next step, a correlation analysis was carried out. A positive, moderately strong relationship was found between AGF and the number of BED symptoms (*rho* = 0.45; *p* <.001). A similar relationship was observed between AGF and general anxiety (*r* =.48; *p* <.001), and a slightly weaker, positive relationship between anxiety and the number of BED symptoms (*rho* = 0.35; *p* <.001). The severity of BED symptoms correlated only with depression, but the relationship was weak and negative (*rho* = − 0.20; *p* <.05). The results of the analyses discussed above are presented in Table 2.

**Table 2:** Spearman’s rank correlations between variables for the entire sample.

Variables	Age	BMI	Severity of BED symptoms	Number of BED symptoms (0–8)	Anxiety About Getting Fat (AGF)	Anxiety	Depression
Age	–						
BMI	0.39***	–					
Severity of BED symptoms	− 0.05	0.20*	–				
Number of BED symptoms (0–8)	− 0.13	− 0.03	0.22*	–			
Anxiety about getting fat (AGF)	− 0.16	− 0.10	0.12	0.46***	–		
Anxiety	− 0.02	− 0.12	0.18	0.35***	0.48***	–	
Depression	0.03	− 0.03	− 0.20*	− 0.14	− 0.12	− 0.23*	–

* *p* <.05; ** *p* <.01; *** *p* <.001.

### Relationships of severity of BED symptoms with depression, anxiety and anxiety about getting fat

A Kruskal-Wallis test compared three groups with mild, moderate, and severe BED symptoms. The results showed that these groups differed significantly in the severity of depression (*F*(2) = 5.55; *p* =.005). The effect size was moderately strong^22^. Post hoc tests were performed to determine the sources of these differences. The results showed that the group with mild BED symptoms (1 to 3 episodes a week) differed in depression severity from the group with moderate BED symptoms (4 to 7 episodes a week). Individuals with mild BED symptoms had more severe depression (*M* = 18.35; *SD* = 3.09), *p* =.005, than those with moderate BED symptoms (*M* = 16.03; *SD* = 3.69). The remaining results did not point to any significant differences among the groups. The means, standard deviations, *F* values, and Dunnett’s T3 multiple comparisons post-hoc tests for each group are shown in Table 3.

**Table 3:** Comparisons of anxiety about getting fat (AGF), anxiety, and depression across BED severity groups.

Variables	Mild BED symptoms (1) *N* = 66		Moderate BED symptoms (2) *N* = 29		Severe BED symptoms (3) *N* = 8		H	*p*	η ^2^	post hoc
	M	SD	M	SD	M	SD				
Anxiety About Getting Fat (AGF)	49.73	16.36	54.28	11.16	55.63	11.22	1.30	0.28	0.03	
Anxiety	16.38	3.43	17.83	3.58	17.25	3.99	1.77	0.18	0.03	
Depression	18.35	3.09	16.03	3.69	18.75	2.76	**5.55**	**0.005**	**0.10**	1 > 2

Post hoc analysis was carried out using Dunnett’s T3 test; coefficients in bold represent statistically significant relationships.

### Relationships of diversity of BED symptoms with depression, anxiety, and anxiety about getting fat

A cluster analysis of the number of BED symptoms in the sample was carried out. We used a two-step cluster analysis approach with hierarchical cluster analysis followed by k-means analysis. Hierarchical cluster analysis was used to determine the optimal solution for the number of clusters. The hierarchical clustering results suggested that the two clusters showed significant differences and adequately represented the data. Accordingly, k-means clustering was performed with the number of clusters set to two. The results of the analysis of differences between the two clusters are presented in Table 4.

**Table 4:** Differences in depression, anxiety, and anxiety about getting fat between a group with a larger number of BED symptoms and a group with a smaller number of BED symptoms.

Variables	Group 1 (Me = 5)			Group 2 (Me = 7)			Statistics	*p*	Effect size
	*N*	M	SD	*N*	M	SD			
BMI	42	27.94	7.05	61	27.70	9.07	1424.50	0.34	0.11
Anxiety About Getting Fat (AGF)	42	39.14	13.27	61	59.95	8.51	242.00	**0.001**	**−0.81**
Anxiety	42	14.05	1.90	61	18.79	3.09	232.00	**0.001**	**−0.82**
Depression	42	18.36	3.36	61	17.30	3.36	1545.00	0.08	0.21

The effect size statistics for the Mann-Whitney U test was the rank biserial correlation; coefficients in bold indicate statistically significant relationships; group 1 – lower diversity of BED symptoms, group 2 – higher diversity of BED symptoms.

Cluster analysis yielded two groups: group 1 with a median number of 5 BED symptoms (lower diversity of BED symptoms) and group 2 with a median number of 7 symptoms (higher diversity of BED symptoms). The Mann-Whitney U test showed that the two groups differed statistically significantly in the mean anxiety score, *U* = 232, *p* <.001. Women with more diverse BED symptoms had a higher mean anxiety score (*M* = 18.79; *SD* = 3.09) than women with less diverse symptoms (*M* = 14.05; *SD* = 1.90). The effect size obtained by using rank biserial correlation showed that the effect was strong^23^. The same difference was observed for the mean AGF, *U* = 242, *p* <.001. Women with a larger number of BED symptoms had a higher mean AGF (*M* = 59.95; *SD* = 8.51) than women with fewer symptoms (*M* = 39.14; *SD* = 13.27), and the effect size was also strong. The detailed results of the analyses are shown in Table 4.

### Prediction of BED symptom diversity based on depression, anxiety, and anxiety about getting fat

Linear regression analysis was performed to test the predictability of BED symptom diversity among the participants. Three predictors were entered into the model: depression, anxiety, and AGF. The explained variable in the model was the number of BED symptoms. The model fitted the data well, *F*(3;102) = 12.19; *p* <.001; *R*^*2*^_adj_. = 0.25 and explained 25% of the variance in the explained variable. In the model AGF β = 0.44, *t* (4.52); *p* <.001 was significant predictor, while depression and anxiety turned out to be non-significant. The estimated value of the regression coefficient indicates that an increase in the intensity of AGF may lead to a rise in the number of BED symptoms. The detailed results of the analyses are shown in Table 5.

**Table 5:** Prediction of BED symptom diversity based on depression, anxiety, and anxiety about getting fat – linear regression analysis.

	B	SE	β	t	*p*
*F*(3;102) = 12.19; *p* <.001 *R*^2^_adj_. = 0.25					
(Constant)	2.48	1.33		1.87	**0.07**
**Anxiety**	**0.07**	**0.05**	**0.13**	**1.30**	**0.20**
Depression	− 0.02	0.05	− 0.04	− 0.49	0.63
Anxiety About Getting Fat (AGF)	0.06	0.01	0.44	4.52	**< 0.001**

Dependent variable: number of BED symptoms; predictors: (Constant), Anxiety, Depression, Anxiety About Getting Fat.

## Discussion

The primary object of the present study was to examine depression and anxiety among women who met the criteria for BED. As hypothesized, BED was associated with both depression and anxiety, but not in a straightforward manner. The diversity of BED symptoms (as measured by the number of those symptoms) was positively associated with anxiety but not with depression. In contrast, BED severity (as measured by the frequency of binge eating episodes) was negatively associated with depression but not with anxiety. In this present study, we observed a difference in anxiety between groups with fewer and more diverse BED symptoms. Women who reported more BED symptoms displayed higher levels of anxiety than women with fewer symptoms. We assume this result with caution. The positive relationship between anxiety and BED was previously reported by other researchers who found that people with higher levels of anxiety experienced more severe binge eating symptoms^24–26^. According to some researchers, certain forms of overeating, including BED, may help individuals regulate negative emotions, such as anxiety, and cope with them more effectively^27,28^. This seems to primarily affect women, as research suggests that anxiety disorders are more common and more severe among women than among men. Furthermore, among women, anxiety disorders are more often associated with depression and eating disorders^29^. The scarcity of studies examining men with BED makes generalizing conclusions difficult. However, one study found that anxiety disorders were the most common comorbid disorder among men with BED^30^. This allows us to apply this result to men as well.

We also found a positive relationship between anxiety and AGF. AGF is associated with body image dissatisfaction and contributes to the development of eating disorders^13^, similar to general anxiety, whose association with eating disorders has been widely described in the literature^6,8,9^. We can cautiously assume that AGF may result from a general tendency to react with anxiety. Moreover, AGF may come as a result of prejudice and stigmatization of overweight and obesity from the environment. Experiences of stigmatization may contribute to the development of anxiety disorders^21^. In the present study, no relationship was observed between AGF and depression. In contrast to Styk et al.‘s study^21^, in which depression, like anxiety, was associated with AGF, our data did not show the existence of this relationship. Perhaps the difference in the findings was due to the type of sample. While in the present study, we investigated a sample that consisted exclusively of women who met the criteria for BED, Styk et al.^21^ examined a non-clinical, gender-diverse study group.

Preliminary evidence suggests that AGF may predict BED^16^. This assumption is supported by the results obtained. AGF was associated with a greater number of BED symptoms, suggesting it may contribute to symptom escalation. This finding is supported by one of the latest studies, which found that anxiety was the strongest predictor of BED occurrence among patients after bariatric surgery, which included both women and men^31^. AGF may be increased by general anxiety disorders, which are common in eating disorders. Some individuals who are intensely preoccupied with the appearance of their own body may experience psychological discomfort, which they try to relieve by eating. Conversely, overeating may reinforce fears of weight gain, which may lead to a vicious cycle of increasing anxiety and coping with it through binge eating.

Unlike the diversity of symptoms, the severity of BED was not associated with anxiety or AGF. It can, therefore, be assumed that anxiety and AGF are associated with the number of BED symptoms but not with the frequency of recurrence of binge eating episodes.

The association between depression and the severity of BED was weak and negative. Further analysis showed that women with mild BED symptoms (1–3 binge eating episodes per week) showed significantly higher levels of depression compared to women with moderate BED symptoms (4–7 binge eating episodes per week). The result obtained contradicts most studies, which indicate a positive association between BED and depression. However, there are also indications that depression is not predictive of binge eating disorder^32^. Research indicates that depression can lead to both increased and decreased appetite, reflecting different patterns of neural activity. Patients with major depressive disorder show significant heterogeneity in appetite, and some of them experience a loss of desire to eat^33^. Based on the obtained results, we can assume that more severe depression may reduce the desire to eat. Several studies suggest the comorbidity of BED with atypical depression, which is additionally associated with loss of appetite and distorted body image^34^. Moreover, there is research that confirms that atypical depression can be considered a subtype of major depression^35^. This may be a possible explanation for the obtained results. It is worth noting that, according to some studies, women report more severe symptoms of depression related to appetite disorders than men^36^. This may be a feature of depression that is more specific to women. Also, we can assume that in the study group, BED and depression co-occurred but were not mutually related. The unequal size of the mild, moderate, and severe groups of severity of BED may have confounded the measurement, because of the underrepresented group of severe BED.

Based on the results obtained, it can be assumed that anxiety and AGF are vital to the diversity of BED symptoms but not for the frequency of binge eating episodes. Similarly, depression may be more vital to the frequency of recurrence of BED episodes than to the number of symptoms. It should be emphasized that all the associations with the diversity of BED symptoms were stronger than the associations with the severity of BED, and the results refer to women. Thus, it can be assumed that the diversity of BED symptoms may play a greater role in the psychopathology of BED than the recurrence rate of binge eating episodes in women, which is supported by our previous research^17^. More BED symptoms may impact various areas of daily life, worsening overall quality of life. Engagement in behaviors linked to BED can serve as an indicator of the severity of binge eating disorder. A greater number of BED-specific behaviors may correlate with increased preoccupation with the disorder and, consequently, with a lower quality of life. This highlights the importance of addressing areas of life that are impaired by BED during diagnosis and treatment. Few studies describe BED from two perspectives: the number of symptoms and the recurrence rate of episodes.

## Strengths and limitations

The key strength of the study is its use of two different perspectives on BED, including the number of symptoms and the recurrence rate of episodes. Most previous studies have focused on how often BED episodes occur, while this study included data on a broader definition of binge eating disorder. Additionally, there is little research on the anxiety about gaining weight among women who binge eat.

Despite our efforts, we could not avoid certain limitations. The final number of participants in the study was relatively small because we aimed to gather a homogeneous sample. Therefore, the groups were not proportionally divided by severity, which affects statistical power and generalizability. Individuals with severe BED symptoms were underrepresented due to limited access to potential participants, as the number of patients with severe BED is generally lower.

The sample consisted only of women because the number of men who met the study criteria was too small, and they had to be excluded, which, of course, increased the risk of gender bias. Further research with a larger sample that includes women, men, and non-binary individuals is necessary to compare the relationships we examined across different genders.

The questionnaire used to assess the presence and severity of BED symptoms has not been standardized or normalized, which impacts the generalizability of the findings. Therefore, caution should be taken when interpreting the results. However, the items’ alignment with the DSM-5 diagnostic criteria ensures the reliability of the data collected. Future research aims to develop a standardized tool for measuring BED.

A limitation of the present study is the absence of systematic correction for multiple testing across the analyses performed. This decision was motivated by the exploratory aim of the study and the relatively small sample size, which could increase the likelihood of Type II errors under strict correction procedures. Nonetheless, the lack of adjustment increases the risk of Type I error, and future research on larger samples should apply appropriate procedures, such as family-wise error rate or false discovery rate control.

Another limitation is related to the cluster analysis. Although it provided interesting insights into the possible differentiation of participants by the number of BED symptoms, the small sample size and unequal cluster sizes reduce the robustness of the findings. Median splits and k-means clustering in small samples may inflate effect sizes. Therefore, the results of this analysis should be treated as exploratory and require replication in larger, more diverse cohorts.

An additional limitation was that the study was cross-sectional and measured the severity of BED at a specific point in time (the participants noted the symptoms that they suffered during the last month); therefore, conclusions about the prediction of BED by AGF should be drawn with caution.

Potential confounding variables, such as coexisting mental disorders, medication use, or treatment status, were not sufficiently controlled, which may have distorted the observed relationships. Future studies should be supplemented with an in-depth clinical interview and access to medical records. Additionally, self-reporting of BMI could be biased due to participants’ distorted body image. In future investigations, the participants’ height and weight should be measured independently to avoid this risk.

## Conclusions

Participants who met the criteria for BED, as indicated by the BED Symptoms Scale used in the study, experienced high levels of anxiety and AGF. Having more BED symptoms was linked to greater severity of both variables. Furthermore, we found that AGF may increase the number of BED symptoms. Participants who met the criteria for BED and engaged in binge eating the least often showed significantly more severe depression symptoms than women who binge ate at a moderate frequency. As depressive symptoms increased, the frequency of BED episodes decreased. The results suggest that the diversity of BED symptoms, measured by their number, may play a more important role in the psychopathology of BED than the recurrence rate of binge eating episodes. This finding is supported by our previous research^17^. Few studies on BED examine the two separate factors of the number of symptoms and the recurrence rate of episodes. Our proposed approach is innovative and worthy of further examination. The findings may offer essential insights for developing effective strategies to prevent and treat BED.

## Data Availability

The data that support this study’s findings are not openly available due to reasons of sensitivity, but are available from the corresponding author upon reasonable request. The data are located in controlled-access data storage at the corresponding author.

## Abbreviations

AGF: Anxiety about getting fat
BED: Binge eating disorder
BMAS: Body Mass Anxiety Scale
BMI: Body mass index
HADS: Hospital Anxiety and Depression Scale
